# Should mathematics teachers be humorous? A study based on the instructional humor processing theory

**DOI:** 10.3389/fpsyg.2025.1668887

**Published:** 2025-12-11

**Authors:** Tommy Tanu Wijaya, Min Feng, Yiming Cao, Mingyu Su, Akhmad Habibi

**Affiliations:** 1 School of Mathematical Sciences, Beijing Normal University, Beijing, China; 2 School of Mathematics and Information Engineering, Longdong University, Qingyang, China; 3 Universitas Jambi, Jambi, Indonesia

**Keywords:** SEM, humor, secondary school, student engagement, student teacher relationship

## Abstract

Teacher humor is considered an important factor in mathematics classroom management for improving student learning outcomes and engagement. The aim of this study was to investigate the relationships between teacher humor (mathematics-related humor, mathematics-unrelated humor, self-disparaging humor, and other-disparaging humor) and student engagement, with teacher–student relationships as a moderator. The respondents were 326 secondary school students, and the analysis was conducted using PLS-SEM. The results showed that six out of seven hypotheses were significant. Specifically, mathematics-related humor and other-disparaging humor had significant positive direct effects on teacher–student relationships and significant indirect effects on student engagement. In contrast, mathematics-unrelated humor had a significant negative effect on teacher–student relationships. These findings suggest that mathematics teachers can effectively use appropriate humor styles to strengthen teacher–student relationships and enhance student engagement in the mathematics classroom.

## Introduction

The success of teaching and learning activities in mathematics is closely tied to effective classroom management (Kim Dong Seok and Seok, 2022; St-Amand et al., 2024). Classroom management remains a significant challenge for mathematics teachers, as it involves complex factors beyond implementing simple classroom rules (Hatch, 1987). One crucial aspect, practically speaking, is managing the classroom in a way that removes barriers between teachers and students, enhances students’ learning motivation and engagement, promotes effective learning, and fosters student cooperation during teaching and learning activities (An and Childs, 2023; Zhan et al., 2021). This study focuses on teachers’ use of humor (e.g., course-related humor, course-unrelated humor, self-disparaging humor, and other-disparaging humor) (Tsukawaki and Imura, 2020) and its relationship with teacher–student relationships and student engagement.

The effectiveness of humor used by teachers in the classroom on student-related learning behaviors and outcomes has been widely explored in previous studies, from elementary to higher education levels (Davies, 2015; Lu'mu et al., 2023; St-Amand et al., 2024). Generally, humor is defined as a message intended to elicit laughter and entertain through unique meanings (Lu'mu et al., 2023). It can be categorized into various types, and its effectiveness in learning contexts has been the focus of much research (Lu'mu et al., 2023). Prior studies have shown that the use of humor in the classroom can create a more enjoyable learning atmosphere, reduce students’ stress levels, and improve their attitudes toward learning (Davies, 2015; Frymier et al., 2008; Van Praag et al., 2017).

This issue becomes particularly interesting in the context of mathematics, a subject often feared by students and associated with high levels of mathematics anxiety (Caviola et al., 2019; Wijaya et al., 2025). Moreover, mathematics teachers are often perceived as having a monotonous and unengaging teaching approach, with mathematics classes frequently viewed as serious and tense environments (Abdullah et al., 2020; Sharma, 2018). Although humor has been widely recognized in general education research as an effective way to promote positive classroom interactions and enhance student engagement, its specific role in mathematics education remains underexplored. Few studies have examined how different types of humor used by mathematics teachers influence teacher–student relationships and students’ emotional and behavioral engagement. This lack of empirical evidence highlights the need for focused research to determine whether and how humor can serve as a meaningful pedagogical strategy in mathematics teaching.

In China, in 2024, the government issued a guideline aimed at developing a high-quality professional teaching force, focusing on improving the quality and innovation of teaching methodologies (Gong et al., 2023; Wu and Shen, 2024). Teacher humor is implicitly included in this guideline, as it emphasizes the need for student-centered and joyful teaching, which encourages the use of humor to foster a positive learning environment. However, there is still a lack of research in China examining whether humor is related to teacher–student relationships and student engagement. If such a relationship exists, what types of humor should mathematics teachers use? These two questions represent both the novelty and the purpose of this study.

### Literature review and hypotheses development

This study adopts the Relational Process Model of Humor (RPMH) to analyze the relationship between teacher humor and the quality of teacher–student relationships (Cooper, 2008). According to this model, humor serves as a social tool that can create emotional closeness, reduce tension, and foster trust between teachers and students. In this sense, humor is not only an expression of personality but also an instructional behavior that.

In the current curriculum, joyful teaching emphasizes student-centered learning environments where emotional connection and enjoyment support active participation and deep understanding. Within this framework, humor can be understood as both a core element and a supportive tool of joyful teaching. It becomes a core element when teachers deliberately use humor to create a lively and positive atmosphere that makes mathematics more approachable. For example, a teacher who uses playful jokes or funny math examples integrates humor as part of the teaching process itself. At the same time, humor also functions as a supportive strategy when it is used to relieve stress, encourage shy students to participate, or strengthen bonds after challenging tasks.

Previous research guided by the Instructional Humor Processing Theory (IHPT) has examined the effects of humor on students’ attitudes and learning outcomes in general education contexts (Lu'mu et al., 2023; St-Amand et al., 2024; Tsukawaki and Imura, 2020; Wanzer et al., 2010). These studies show that humor can have both positive and negative effects depending on its type and students’ interpretation. However, despite the growing recognition of humor as a valuable instructional strategy, empirical research on humor in mathematics education remains limited. Most existing studies focus on general classroom communication or language teaching, leaving a gap in understanding how humor operates in subjects that are often perceived as difficult and anxiety-inducing, such as mathematics.

Mathematics classes are frequently characterized by a serious and high-pressure learning environment, where students experience considerable stress and fear of failure. In such contexts, humor may play a unique role in transforming classroom relationships and improving engagement, yet this potential has not been systematically examined. To address this gap, the present study investigates how different types of humor used by mathematics teachers influence teacher–student relationships and student engagement. By focusing on the mathematics classroom context, this study contributes to extending humor theories such as RPMH and IHPT into a subject area where emotional and relational factors are often overlooked.

### Perceived related humor and teacher–student relationship quality

Humor can be defined as a form of entertainment that elicits laughter and enjoyment (Davies, 2015). In educational settings, humor has been shown to break the silence, ease serious classroom atmospheres, and create a more relaxed and enjoyable learning environment (Koç, 2023; Tobin et al., 2013). By using humor, teachers can reduce students’ stress levels, alleviate boredom with the subject matter, and “break the ice” in the classroom (Erdoğdu and Çakıroğlu, 2021). Moreover, humor is often used as a strategy to build positive relationships (Erdoğdu and Çakıroğlu, 2021; Şahin, 2021; Van Praag et al., 2017). Therefore, we believe that the use of humor by mathematics teachers in the classroom can strengthen the relationships between teachers and students.

Theoretically, the relationship between humor and relationship quality is explained by the Relational Process Model of Humor (RPMH) (Cooper, 2008). This model suggests that positive relationships can be fostered through the use of humor, which promotes closeness and improves interpersonal interactions. Previous studies have also demonstrated the link between humor and relationship quality in workplace contexts (Huber, 2022; Zhang et al., 2022). For example, leaders who frequently use humor positively influence employees’ behavior and enhance the quality of workplace relationships (Wijewardena et al., 2024). Drawing on these insights, we suggest that similar dynamics may apply in educational contexts, where teacher humor could help build stronger bonds with students.

**Figure 1:**
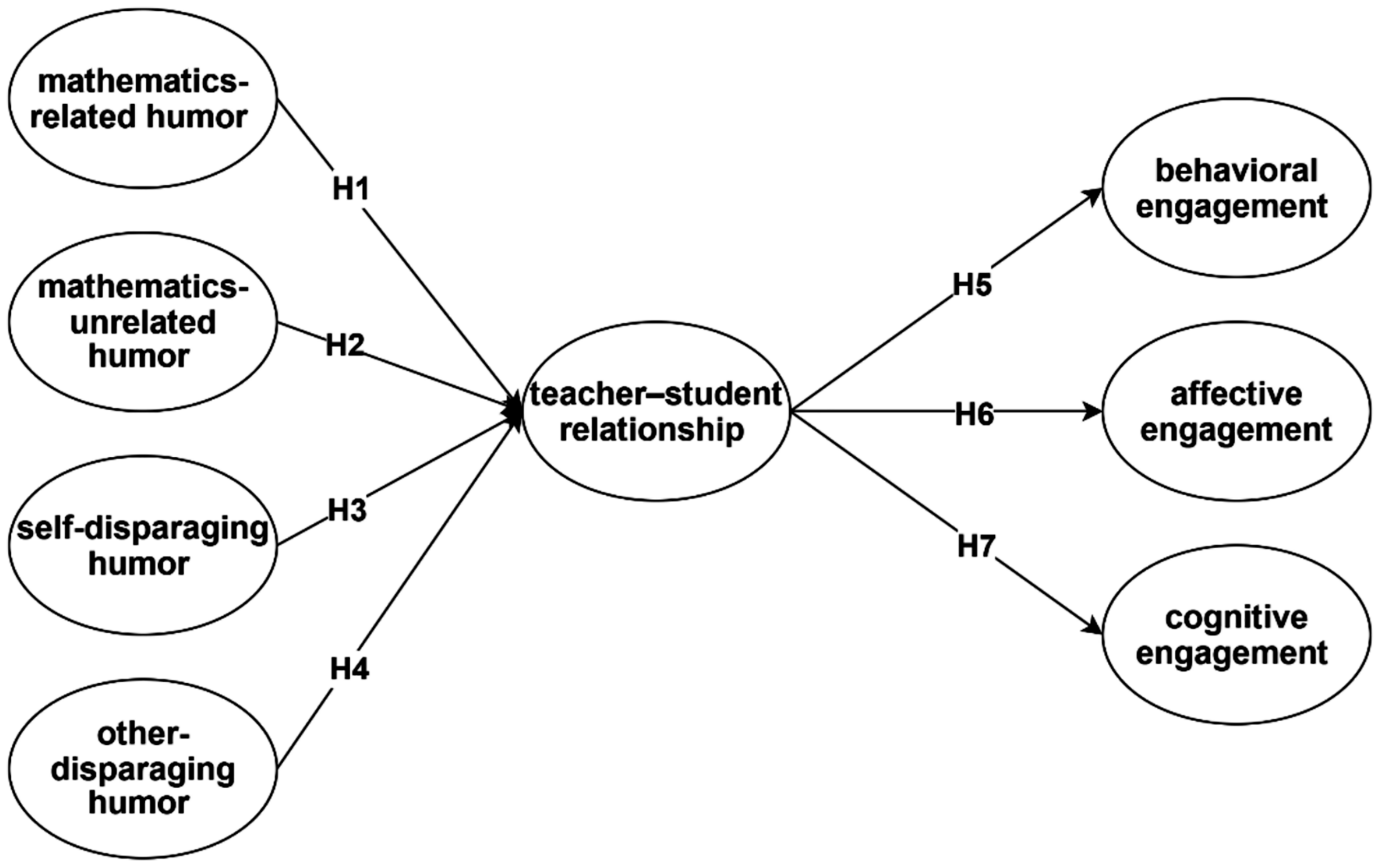
Conceptual framework.

All types of humor, when used appropriately, may contribute to these positive outcomes (Tsukawaki and Imura, 2020). Mathematics-related humor (MRH) directly connects to lesson content, making learning more engaging and reinforcing students’ perception of their teacher as approachable and supportive. Mathematics-unrelated humor (MUH), though not tied to the content, can lighten the atmosphere and help build rapport by showing the teacher’s personality and approachability. Self-disparaging humor (SDH) may humanize teachers, making them appear more relatable and approachable to students. Finally, other-disparaging humor (ODH), when applied sensitively and not directed at students, can foster a sense of shared amusement and strengthen the classroom community. Drawing from these theoretical perspectives and empirical findings, we posit that all four types of humor used by mathematics teachers are positively associated with the quality of teacher–student relationships (Figure 1).

*H1*: Mathematics-related humor (MRH) is positively associated with teacher–student relationships.

*H2*: Mathematics-unrelated humor is positively associated with teacher–student relationships.

*H3*: Self-disparaging humor is positively associated with teacher–student relationships.

*H4*: Other-disparaging humor is positively associated with teacher–student relationships.

### Teacher student relationship and student engagement

Teaching and learning are inherently interactive processes between teachers and students (Lu'mu et al., 2023). It is widely recognized that a teacher’s ability to build strong relationships with their students is a key factor in achieving effective teaching and learning in the classroom (Choi Kwon et al., 2013; Trice et al., 2023; Zhao et al., 2024). This study proposes the initial hypothesis that teacher–student relationships are critical for fostering student engagement in mathematics learning activities. The quality of the relationship between teachers and students is characterized by trust and respect arising from social interactions, which contribute to intrinsic motivation and positive student perceptions of their teachers (Hagenauer et al., 2015; Pianta et al., 2012). Based on this reasoning, we hypothesize that teacher–student relationships are positively associated with student engagement in mathematics classrooms.

*H5*: teacher student relationship positively associated with behavioral engagement

*H6:* teacher student relationship positively associated with affective engagement

*H7*: teacher student relationship positively associated with cognitive engagement

## Methodology

### Research design

We employed a cross-sectional survey design (Forero et al., 1999) to investigate teacher humor (mathematics-related humor, mathematics-unrelated humor, self-disparaging humor, and other-disparaging humor) and student engagement, with teacher–student relationships as a moderator. While cross-sectional designs cannot precisely determine causal linkages, they are valuable for examining complex interactions among latent variables. This study included a sample size of over 300, which meets the recommended threshold for SEM analyses with factor loadings above 0.60 (Hair et al., 2019). PLS-SEM was applied to effectively manage and assess complex predictive associations within the structural model (Bayaga, 2024).

### Measures

The data for this study were collected using eight validated scales measuring affective engagement (AE), behavioral engagement (BE), cognitive engagement (CE), mathematics-related humor (MRH), mathematics-unrelated humor (MUH), other-disparaging humor (ODH), self-disparaging humor (SDH), and teacher–student relationship (STR). Each construct was assessed using a 5-point Likert scale ranging from 1 (“strongly disagree”) to 5 (“strongly agree”). A pilot test with 50 participants confirmed the clarity and suitability of the instrument. Only minor wording modifications were made to finalize the questionnaire.

### Student engagement

Student engagement in this study was originally based on the conceptual framework proposed by Barkatsas et al. (2009), Pietarinen et al. (2014) which conceptualizes student engagement as comprising affective engagement (AE), behavioral engagement (BE), and cognitive engagement (CE). During the pilot test, the scale achieved a Cronbach’s alpha of 0.86, indicating strong internal consistency.

### Teacher student relationship

The teacher–student relationship instrument used in this study was adapted from Graen and Scandura (1987) and Sulkowski and Simmons (2018) focusing on the emotional aspects of the teacher–student relationship. In the pilot study, the scale achieved a Cronbach’s alpha of 0.88, indicating strong internal consistency. The complete instrument can be found in Appendix 1.

### Teacher humor

The teacher humor questionnaire used in this study was adapted from the typology of humor, which classifies humor into four categories: mathematics-related humor (MRH), mathematics-unrelated humor (MUH), other-disparaging humor (ODH), and self-disparaging humor (SDH) (Leist and Müller, 2013; Lu'mu et al., 2023; St-Amand et al., 2024; Van Praag et al., 2017). The questionnaire consisted of a total of 14 items. Reliability testing in this study yielded a Cronbach’s alpha of 0.89, indicating excellent internal consistency.

## Participants

The sample for this study consisted of secondary school students from four schools (see Table 1). A total of 326 responses were successfully collected using a convenience sampling method, as these schools agreed to support the research. Among the students who voluntarily completed the questionnaires, 159 were male (48.8%) and 167 were female (51.2%), with 142 students from Grade 10 (43.6%), 161 from Grade 11 (49.4%), and 23 from Grade 12 (7.1%). Regarding their perceptions of the mathematics teacher–student relationship, 49 students (15.0%) rated it as “very good,” 128 (39.3%) as “good,” 135 (41.4%) as “normal,” 11 (3.4%) as “bad,” and 3 (0.9%) as “very bad.” The study protocol was approved by the School of Mathematical Sciences, Beijing Normal University, and informed consent was obtained from all participating students.

**Table 1:** Demographic characteristics of secondary school students.

Characteristics	Level	*N*	%
Gender	Male	159	48.8
	Female	167	51.2
Grade	Grade 10	142	43.6
	Grade 11	161	49.4
	Grade 12	23	7.1
Teacher student relationship	Very bad	3	0.9
	bad	11	3.4
	normal	135	41.4
	good	128	39.3
	Very good	49	15

## Data collection process

We collected the data using paper-based forms after obtaining approval from Beijing Normal University and the four target schools. Before students began filling out the questionnaire, their mathematics teachers informed them that participation was anonymous, confidential, and entirely voluntary, with the data used solely for research purposes. After providing signed consent forms, the respondents proceeded to complete the questionnaires, which on average took 15–25 min. The completed paper-based surveys were then placed into envelopes, sealed, and sent back to the research team for analysis.

## Data analysis and results

We analyzed the data using SmartPLS 4.0 to apply Partial Least Squares Structural Equation Modeling (PLS-SEM) (Hair et al., 2016). PLS-SEM was chosen over covariance-based SEM (CB-SEM) because our data showed a non-normal distribution and the sample size was relatively small (Yuan et al., 2023). In such conditions, PLS-SEM is often recommended as it offers greater statistical power and robustness (Gao et al., 2025). Moreover, PLS-SEM is particularly well-suited for studies that involve model development or extension, as well as complex structural models with moderating effects (Qin et al., 2022). In this study, for instance, we examined teacher–student relationships as a moderator between teacher humor and student engagement, making PLS-SEM an appropriate choice.

## Common method bias

Since our study relied on self-reported data from respondents, we considered it important to address potential common method bias (CMB) to ensure the validity of our findings (Wijaya et al., 2024). At the procedural level, the questionnaire was distributed anonymously, and all students were informed that their participation was entirely voluntary and without any pressure. At the statistical level, we examined collinearity using Variance Inflation Factor (VIF) values as suggested by Hair et al. (2019), where VIF values below 5.0 indicate no serious threat to validity. In our analysis, the VIF values ranged from 1.058 (ODH4) to 2.626 (CE2), which are well within acceptable limits. These results provide reassurance that collinearity does not pose a major concern, and common method bias is unlikely to have significantly influenced our findings.

## Measurement model analysis

The measurement model in this study was evaluated using Cronbach’s alpha, composite reliability, and average variance extracted (AVE). As shown in Table 2, all constructs demonstrated Cronbach’s alpha values above 0.80 and AVE values exceeding 0.50, indicating that all constructs in this study have strong internal consistency and satisfactory convergent validity (Hair et al., 2019).

**Table 2:** Measurement model analysis (reliability and validity).

Construct	Item	Factor loadings	Cronbach’s alpha	Composite reliability (rho_a)	Composite reliability (rho_c)	Average variance extracted (AVE)
AE	AE1	0.812	0.827	0.853	0.883	0.655
	AE2	0.852				
	AE3	0.828				
	AE4	0.739				
BE	BE1	0.818	0.850	0.852	0.899	0.690
	BE2	0.811				
	BE3	0.879				
	BE4	0.813				
CE	CE1	0.862	0.862	0.870	0.916	0.784
	CE2	0.918				
	CE3	0.876				
MRH	MRH1	0.798	0.788	0.794	0.863	0.611
	MRH2	0.762				
	MRH3	0.818				
	MRH4	0.748				
MUH	MUH1	0.749	0.779	1.819	0.812	0.599
	MUH2	0.771				
	MUH3	0.962				
ODH	ODH1	0.734	0.717	0.825	0.876	0.671
	ODH2	0.743				
	ODH3	0.742				
	ODH4	0.942				
SDH	SDH1	0.754	0.716	0.837	0.832	0.626
	SDH2	0.896				
	SDH3	0.710				
STR	STR	0.881	0.842	0.842	0.905	0.760
	STR2	0.873				
	STR3	0.861				

For discriminant validity, we referred to both the Fornell-Larcker criterion and the Heterotrait-Monotrait ratio (HTMT) (Fornell and Larcker, 1981). As shown in Table 3, the square root of the AVE for each construct was higher than its correlations with other constructs, satisfying the Fornell-Larcker criterion. Additionally, Table 4 shows that all HTMT values were below the threshold of 0.85, indicating that no constructs demonstrated problematic overlap. These results confirm that each construct in this study is unique and distinct, supporting the discriminant validity of the measurement model.

**Table 3:** Fornell Larcker criterion (discriminant validity).

Construct	AE	BE	CE	MRH	MUH	ODH	SDH	STR
AE	0.809							
BE	0.741	0.831						
CE	0.720	0.738	0.886					
MRH	0.558	0.481	0.436	0.782				
MUH	−0.060	−0.054	−0.016	−0.048	0.774			
ODH	0.348	0.340	0.306	0.319	−0.133	0.520		
SDH	0.189	0.122	0.156	0.177	0.431	0.104	0.791	
STR	0.600	0.514	0.425	0.458	−0.164	0.465	0.085	0.872

**Table 4:** HTMT (discriminant validity).

Construct	AE	BE	CE	MRH	MUH	ODH	SDH	STR
AE								
BE	0.786							
CE	0.783	0.762						
MRH	0.679	0.587	0.525					
MUH	0.085	0.084	0.052	0.148				
ODH	0.328	0.299	0.262	0.314	0.696			
SDH	0.232	0.143	0.191	0.218	0.683	0.719		
STR	0.696	0.607	0.497	0.556	0.135	0.410	0.111	

## Structural model analysis

**Figure 2:**
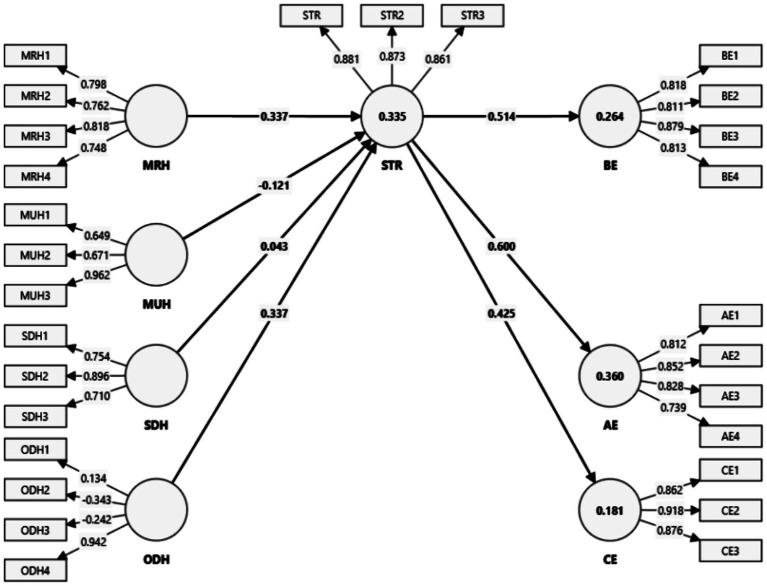
Final model with R2, path coefficient and loadings.

For the structural model analysis, we applied 5,000 bootstrap resamples, and hypothesis testing was conducted at a 95% confidence level, with *p*-values below 0.05 indicating significant relationships (Zhang et al., 2024). As shown in Figure 2, the structural model demonstrates explanatory power across four key outcomes. Specifically, it explains 33.5% of the variance in teacher–student relationships, 26.4% in behavioral engagement, 36% in affective engagement, and 18.1% in cognitive engagement. According to Hair et al. (2019), these R^2^ values indicate moderate explanatory power for teacher–student relationships, behavioral engagement, and affective engagement, and acceptable explanatory power for cognitive engagement, which is reasonable given the exploratory nature of this study.

The results of the hypothesis testing are presented in Table 5, showing that six out of seven hypotheses were statistically significant. Hypothesis 1 revealed that mathematics-related humor (MRH) has a significant positive relationship with teacher–student relationships (*β* = 0.337, *p* < 0.001), indicating that when teachers use humor directly connected to mathematics content, it strengthens the relationship between teachers and students. Hypothesis 2 found that mathematics-unrelated humor (MUH) has a significant negative relationship with teacher–student relationships (*β* = −0.121, *p* = 0.035), suggesting that frequent use of humor unrelated to the subject matter may weaken the teacher–student relationship. In contrast, Hypothesis 3 showed that self-disparaging humor (SDH) does not have a significant relationship with teacher–student relationships (*β* = 0.043, *p* = 0.486). Hypothesis 4 demonstrated that other-disparaging humor (ODH) has a significant positive relationship with teacher–student relationships (*β* = 0.337, *p* < 0.001), which may reflect how students respond to humor directed at external situations or figures in a way that builds rapport. Finally, Hypotheses 5, 6, and 7 revealed that teacher–student relationships significantly predict student engagement: behavioral engagement (BE) (*β* = 0.514, *p* < 0.001), affective engagement (AE) (*β* = 0.600, *p* < 0.001), and cognitive engagement (CE) (*β* = 0.425, *p* < 0.001). These results suggest that teacher humor is one potential strategy for strengthening teacher–student relationships, which, in turn, enhances student engagement in the mathematics classroom.

**Table 5:** Results of hypothesis testing.

Path	Path coefficients	Mean	Standard deviation	T statistics	*p* values
H1: MRH → STR	0.337	0.335	0.057	5.892	0.000
H2: MUH → STR	−0.121	−0.129	0.057	2.112	0.035
H3: SDH → STR	0.043	0.054	0.061	0.696	0.486
H4: ODH → STR	0.337	0.337	0.087	3.857	0.000
H5: STR → BE	0.514	0.518	0.039	13.233	0.000
H6: STR → AE	0.600	0.604	0.033	18.288	0.000
H7: STR → CE	0.425	0.428	0.041	10.305	0.000

The results indicate that teacher humor has significant indirect relationships with student engagement through the teacher–student relationship (Table 6). Specifically, the analysis shows that mathematics-related humor (MRH) and other-disparaging humor (ODH) have significant positive indirect effects on student engagement through teacher–student relationships (*p* < 0.001). In contrast, mathematics-unrelated humor (MUH) has significant negative indirect effects (*p* < 0.05), while self-disparaging humor (SDH) shows no significant indirect relationship.

**Table 6:** Indirect effect.

Indirect effect	Path coefficients	Sample mean	Standard deviation	T statistics	*p* values
SDH → STR → CE	0.018	0.023	0.026	0.685	0.493
MRH → STR → AE	0.202	0.202	0.037	5.428	0.000
MRH → STR → BE	0.173	0.174	0.034	5.029	0.000
MUH → STR → AE	−0.073	−0.078	0.035	2.083	0.037
MRH → STR → CE	0.143	0.144	0.030	4.859	0.000
MUH → STR → BE	−0.062	−0.067	0.030	2.086	0.037
ODH → STR → AE	0.202	0.203	0.054	3.725	0.000
MUH → STR → CE	−0.051	−0.055	0.026	2.012	0.044
ODH → STR → BE	0.173	0.175	0.047	3.701	0.000
SDH → STR → AE	0.026	0.033	0.037	0.690	0.490
ODH → STR → CE	0.143	0.144	0.040	3.578	0.000
SDH → STR → BE	0.022	0.028	0.032	0.696	0.487

## Discussion

The use of humor by mathematics teachers in teaching and learning activities is an interesting topic that has been studied in various countries (Bishara, 2023; Weber, 2016). This is because many studies have found that humorous teachers have a positive effect on student learning outcomes (Berge and Anderhag, 2025; Ngai et al., 2025; Tianli et al., 2024). Building on this body of research, the present study classifies types of humor and examines their relationships with teacher–student relationships and student engagement from the students’ perspective.

This study further provides new empirical evidence regarding the types of humor that mathematics teachers should use in the classroom. Students indicated that mathematics-related humor (MRH) and other-disparaging humor (ODH) have a significant relationship in maintaining positive teacher–student relationships (H1 and H4). When teachers use humor that links directly to mathematical ideas, students may find the subject more interesting and less stressful. For instance, a teacher might make a light joke about geometry by saying that a triangle refused to attend a party because it had too many angles. Such humor keeps the lesson engaging and helps students see the teacher as friendly and supportive while staying focused on learning. This aligns with the Relational Process Model of Humor (Wanzer et al., 2010), which explains that humor can strengthen interpersonal relationships in this context, between teachers and students. Similarly, Affiliative Humor (Şahin, 2021) emphasizes that humor enhances interpersonal connections. These findings highlight that secondary school students also expect their mathematics teachers to be humorous and engaging.

Interestingly, this study found that secondary school students in China perceive mathematics-unrelated humor (MUH) as having a significant negative impact on teacher–student relationships (H2). When teachers use humor that is not connected to the lesson, such as jokes about daily life or entertainment, students may feel that the teacher is not focused on teaching. For example, if a teacher spends time joking about a funny movie or weekend plans, students might think their valuable learning time is being wasted. As a result, MUH can weaken students’ trust and respect toward the teacher. This finding may reflect the cultural and educational context of Chinese secondary school students, who are preparing for highly competitive college entrance examinations (Liu and Helwig, 2022; Sang et al., 2018). In this environment, mathematics is regarded as a serious subject, and students may view off-topic humor as a distraction that wastes valuable instructional time (Wijaya et al., 2025). Furthermore, self-disparaging humor (SDH) was found to have no significant relationship with either teacher–student relationships or student engagement (H3). Therefore, it is not recommended for teachers to use this type of humor in the mathematics classroom.

Finally, teacher–student relationships were found to successfully moderate the relationship between teacher humor and student engagement (H5–H7). This finding aligns with Self-Determination Theory (Ryan and Deci, 2000), which emphasizes that the factor of relatedness can enhance student engagement. In the context of this study, relatedness refers to the mathematics teacher–student relationship, which plays a crucial role in fostering a supportive classroom environment that strengthens the impact of teacher humor on student engagement.

### Theoretical and practical implications

This study provides a valuable contribution to the theoretical understanding of mathematics teacher humor, grounded in the Instructional humor processing theory (Tsukawaki and Imura, 2020), with teacher–student relationships examined as a moderating factor. By categorizing types of humor, the study identifies which forms are most effective and significantly enhance teacher–student relationships and student engagement in the mathematics classroom. Additionally, this research offers practical insights for mathematics teachers, helping them to adopt humor strategies that are culturally and contextually appropriate. Methodologically, the inclusion of teacher–student relationships as a moderator extends existing models (St-Amand et al., 2024) and highlights the complex dynamics between teacher behaviors and student engagement in high-stakes educational environments such as Chinese secondary schools.

From a practical perspective, the findings of this study suggest that schools can design teacher training programs that help mathematics teachers use humor more effectively in the classroom. For example, schools can organize professional development workshops where teachers learn how to create mathematics-related humor, such as using funny problem examples, light jokes about common math errors, or humorous stories that connect mathematics to real life. Some existing programs in China, such as local teacher learning communities and national teaching skills workshops, already include modules on classroom communication and emotional climate. These can be expanded to include sessions on humor use, demonstration lessons, and peer observation activities, allowing teachers to practice appropriate humor and receive constructive feedback.

In addition, schools can highlight the importance of maintaining positive teacher–student relationships as an essential element of student engagement. Training can guide teachers to recognize which types of humor build trust and motivation, and which types may cause discomfort or distraction. The findings of this study can therefore serve as a reference for teacher educators and school leaders in developing practical strategies that support harmonious classroom interactions and greater engagement in mathematics learning.

### Limitations

No study is without limitations, and this research is no exception. First, this study relied on self-reported data, which may be subject to potential common method bias and social desirability influences (Holzberger and Prestele, 2021). Future research could address this by incorporating complementary data sources, such as classroom observations or behavioral metrics, to validate self-report findings. Second, as a cross-sectional study, it captures student perceptions at a single point in time, which may not reflect how their responses evolve over time (Gou et al., 2024). Longitudinal studies are therefore recommended to examine these temporal dynamics more comprehensively. Third, cultural differences in humor perception may limit the generalizability of the findings across different countries. We encourage further studies to replicate and test our research model in diverse cultural contexts. Finally, we believe the Instructional humor processing theory could be integrated with other theoretical frameworks in future work to potentially achieve stronger explanatory power for student engagement outcomes.

## Conclusion

Mathematics teachers have employed various strategies to strengthen teacher–student relationships and enhance student engagement in the mathematics classroom. This study provides a comprehensive analysis of how teacher humor contributes to these outcomes, grounded in the Instructional humor processing theory. The findings offer convincing evidence that teacher humor is positively associated with both teacher–student relationships and student engagement. In particular, the use of mathematics-related humor and other-disparaging humor emerged as effective strategies. These insights can inspire mathematics teachers to create a more enjoyable and engaging classroom atmosphere through the thoughtful use of humor.

## Data Availability

The original contributions presented in the study are included in the article/Supplementary material, further inquiries can be directed to the corresponding author.
